# *MYOZ1* Gene Promotes Muscle Growth and Development in Meat Ducks

**DOI:** 10.3390/genes13091574

**Published:** 2022-09-02

**Authors:** Tingting Zhou, Yijing Wu, Yulin Bi, Hao Bai, Yong Jiang, Guohong Chen, Guobin Chang, Zhixiu Wang

**Affiliations:** 1 College of Animal Science and Technology, Yangzhou University, Yangzhou 225009, China; 2 Institute of Animal Husbandry and Veterinary Medicine, Anhui Academy of Agricultural Sciences, Hefei 230031, China

**Keywords:** meat duck, *MYOZ1*, muscle growth and development, single nucleotide polymorphism, protein expression, feed conversion ratio

## Abstract

To explore the effect of *MYOZ1* in the muscle growth and development of meat ducks, *MYOZ1* single-nucleotide polymorphism loci were screened at the DNA level in the meat duck population with highest and lowest feed conversion rates. The expression of *MYOZ1* was detected using reverse-transcription quantitative polymerase chain reaction. The protein expression of MYOZ1 was detected using Western blotting at the protein level. The results showed that there was a base mutation site at 30 bp and 158 bp in the fourth exon of *MYOZ1*, which was mutated from C to T (exon4 C30T) and from G to A (exon4 G158A), respectively. The allele frequency of the locus was significantly different between the high and low feed conversion rate groups (*p* < 0.01). The relative expression of *MYOZ1* mRNA in breast muscle tissue of HF ducks was significantly higher than that of LF ducks (*p* < 0.01). The MYOZ1 protein expression of HF ducks was significantly higher than that of LF ducks (*p* < 0.01). In general, *MYOZ1* has a positive regulatory effect on the muscle growth and development of meat ducks. The results of this study lay a certain theoretical basis for the muscle growth and development of meat ducks.

## 1. Introduction

Muscle growth traits are an important factor affecting economic traits such as the meat production of livestock and poultry, especially the growth traits of skeletal muscle, which are closely related to poultry meat yield [1,2]. Skeletal muscle is composed of different fiber types arranged in a mosaic pattern [3]. Its growth and development are complex processes that involve many regulatory molecules and lead to corresponding reactions and changes in livestock and poultry bodies [4]. In addition to the formation of relatively complex network relationships and signaling pathways, the entire process also involves internal and external environments [4]. As the main component of skeletal muscle, muscle fiber is closely related to its characteristics such as type, quantity, stretchability, and viscosity [5], and the growth and development of muscle fibers is affected by their characteristics, especially the diameter, number, size, and species [6]. In recent years, molecular biotechnology has developed rapidly in various fields. High-throughput sequencing and sequence capture technologies have provided new opportunities for genomics [7]. Therefore, research related to the molecular regulatory mechanisms of muscle growth and development has also significantly progressed. At present, genes with obvious regulatory effects on muscle development include the calsarcin family [8], myostatin (MSTN) [9], the MyoD (MyoD-family protein j) family [10], the MEF2 (myocyte enhancer factor 2) family [11], and the MRF (male recombination factor) family [12]. Using these candidate genes for molecular breeding provides an effective way to improve poultry production traits [13].

Based on the transcriptome sequencing results of the duck F2 resource population constructed earlier by our research group, the *MYOZ1* gene can be a candidate gene that affects the muscle growth traits of meat ducks. *MYOZ1* has great value in the growth and development of skeletal muscle as well as the regulation of muscle fiber types, affecting the animal body composition and meat quality.

To date, the calsarcin family is composed of the *MYOZ1* (*calsarcin-2* or *CS-2*), *MYOZ2* (*calsarcin-1* or *CS-1*), and *MYOZ3* (*calsarcin-3* or *CS-3*) genes. The calsarcin family is a novel family of muscle-specific sarcomeric proteins that link calcineurin to the contractile apparatus [8], thereby potentially promoting the tight connection between muscle activity and activation of calcineurin [14]. Calcineurin (CaN) is a calcium/calmodulin-dependent serine–threonine phosphatase that plays an important role in transducing calcium-dependent signaling in a variety of cell types. The calsarcin family interacts with calcineurin, which is involved in the Ca^2+^ signal transduction pathway and can also act as a substrate of CaN to regulate the expression of skeletal muscle slow muscle fiber genes [15].

*MYOZ1* is mainly expressed in fast-twitch muscle fibers of livestock and poultry skeletal muscle [16], and the calsarcin-2 protein encoded by it can regulate the conversion of skeletal muscle fiber types by regulating the activity of calcineurin [17]. Because *MYOZ1* has an irreplaceable role in signal transduction and muscle fiber type differentiation, it is a potential candidate gene affecting meat quality traits in livestock and poultry [18]. Calcineurin plays an important role in the growth and development of muscle fibers. *MYOZ1* of livestock and poultry inhibits the activity of calcineurin, indirectly acting on the activated T-cell nuclear factor (CaN-NFAT) signaling pathway, thereby preventing the conversion of fast-twitch muscle fibers to slow-twitch muscle fibers [14]. In 2008, Frey et al. reported that *MYOZ1* interacted with the activity of calcineurin and connected calcineurin with α-actinin, and that *MYOZ1* of livestock and poultry also had a direct effect on the diameter of skeletal muscle fibers [19].

The MYOZ1 protein, also known as calsarcin-2 protein or CS-2 protein, is encoded by the *MYOZ1* gene, which is expressed only in fast-twitch muscles and at significantly lower levels in several other tissues [19,20,21]. MYOZ1 is a 299 amino acid nuclear protein that interacts with the Z-disc protein, α-actinin, filamin 2, and PP2B (calcineurin), and effectively forms bridges between proteins and muscle fibers, participating in muscle sarcomere microstructure [22]. Through these interactions, MYOZ1 protein couples striated muscle activity with protein activation and is thought to play a role in both Z-disc assembly and myofibril formation. The calsarcin protein family was confirmed to be specifically located on the muscle Z-line by immunofluorescence experiments [23]. The MYOZ1 protein, as one of the constituent proteins of the Z line, can lead to cardiac hypertrophy and other diseases if the amino acid sequence changes owing to mutation [23]. Since the *MYOZ1* gene is closely related to muscle formation, mutations in the gene encoding calsarcin-2 can be associated with muscular dystrophy and neuromuscular myopathy [24].

In conclusion, *MYOZ1* can negatively regulate the calcineurin/NFAT signaling pathway by inhibiting the activity of calcineurin, thereby indirectly regulating the transformation of muscle cells. It can also change the composition of the fast and slow muscles, leading to changes in the composition of the skeletal muscle. This, in turn, induces changes in function, thereby affecting the appearance and flavor of the muscle. It has important value in the growth and development of skeletal muscle, the regulation of muscle fiber type, and the process of animal signal transmission. The objective of this study is to explore the differences in DNA, RNA, and protein levels between highest feed conversion ratio (HF) and lowest feed conversion ratio (LF) ducks, so as to reveal the functional regulation of the *MYOZ1* gene on the muscle growth and development of ducks, and lay a theoretical foundation for the further improvement of duck breeding.

## 2. Materials and Methods

### 2.1. Animals and Sample Collection 

The test material used in this experiment was the F2 population of the Cherry Valley Duck (CV) × Runzhou Crested White Duck (CC) hybrid duck, which was obtained from Jiangsu Shuyang Zhongke Breeding Poultry Co., Ltd. In accordance with NY/T823-2020 “Poultry Production Performance Terminology and Measurement Statistical Methods”, all ducks were fed under the same feeding conditions, and the feeding mode was ad libitum. Blood was collected from 304 ducks in the F2 population at 42 days of age; the body weight and individual intake were measured to calculate the feed conversion ratio (FCR, feed intake/weight gain). Tissue samples such as muscle were collected and stored at −80 °C. A total of 50 blood samples from meat ducks with highest feed conversion ratio (HF) and 50 meat ducks with lowest feed conversion ratio (LF) were selected, and a total of 100 blood samples were used for gene polymorphism analysis. The muscle tissues of 6 HF ducks and 6 LF ducks were selected for tissue expression analysis at the RNA level. The muscle tissues of 3 HF ducks and 3 LF ducks were selected for protein expression analysis.

### 2.2. DNA and Total RNA Extraction 

In this experiment, the traditional method was used to extract DNA from blood samples of 50 HF individuals and 50 LF individuals. A total of 20 μL of each blood sample was added to 800 μL of lysate and 25 μL of proteinase K (10 mg/mL). The samples were subjected to thermal oscillation for 20 min at intervals of 1 h. The samples were then heated overnight at 55 °C in a water bath. Then, 600 μL of Tris-phenol was added, then the sample was shaken for 10 min before 10 min of centrifuging at 12,000 rpm. Next, 600 μL chloroform was added to the supernatant, and was shaken for 10 min at 12,000 rpm, before being centrifuged for 10 min. The supernatant was then shaken with 1000 μL anhydrous ethanol for 10 min at 12,000 rpm, and centrifuged for 10 min. The DNA was then extracted as a white flocculent precipitation. Following the manufacturer’s instructions, the total RNA was extracted from muscle tissue of 6 HF ducks and 6 LF ducks by using the Trizol reagent kit (Invitrogen, Carlsbad, CA, USA); operation steps can be referenced [25,26]. The quality and concentration of the extracted DNA and RNA were detected using a UV-Vis spectrophotometer. The obtained DNA was stored at −20 °C, while the RNA was stored at −80 °C.

### 2.3. cDNA Synthesis

The total RNA was reverse-transcribed using the FastKing one-step reverse-transcription fluorescence quantitative kit (from Tiangen-KR118, Beijing, China) (see protocol on https://www.tiangen.top/Home/Special/274, accessed on 13 June 2020), and the obtained cDNA was stored at −20 °C for future use.

### 2.4. Reverse Transcription-Quantitative Polymerase Chain Reaction (qRT-PCR)

The *MYOZ1* (accession number XM_005015683) mRNA sequence of ducks in the GenBank was queried, and the fluorescent quantitative primers were designed with the housekeeping gene *GAPDH* (accession number XM_038180584) as the reference gene (Table 1), synthesized by Nanjing Qingke Biotechnology Co., Ltd. (Nanjing, China). Using the cDNA obtained by reverse transcription as the template, the *MYOZ1* and *GAPDH* genes were amplified with the quantitative primers listed in Table 1, and each sample was set to have three replicates. The equipment used was a Light Cycler 96 Real-Time PCR Detection System (Roche, Basel, Switzerland). A total reaction volume of 20 μL was used, including 10 μL of PowerUp SYBR Green Master Mix (A25742, Thermo Fisher, Beijing, China), 8.2 μL of ddH_2_O, 0.4 μL of upstream (10 μM) and 0.4 μL of downstream (10 μM) primers, and 1 μL of cDNA template. Quantitative real-time PCR (qRT-PCR) procedure is as follows: pre-denaturation at 95 °C for 5 min, followed by 40 cycles of denaturation at 95 °C for 10 s and annealing at 60 °C for 30 s; melting curve: 95 °C for 15 s, 60 °C for 60 s, and 95 °C for 15 s. The relative mRNA expression in the samples was calculated using the 2^−Δct^ method. 

### 2.5. Extraction of Sample Protein to Be Tested

Extraction was performed in accordance with the manufacturer’s instructions (Beyotime Biotechnology, Nanjing, China). Individual breast muscle tissues (80 mg) of three HF and three LF meat ducks were weighed, and 600 μL of protein lysis buffer (RIPA buffer:PMSF = 100:1) was added to each tube and homogenized with a glass homogenizer until fully lysed. After complete lysis, the supernatant was centrifuged at 12,000 rpm for 5 min, and the supernatant obtained was used as the sample protein to be tested. BCA standard solution (0.5 mg/mL) was added in the amounts of 0, 1, 2, 4, 8, 12, 16, and 20 μL, respectively, and PBS was added to 20 μL to obtain standard protein solutions with a series of concentrations, which were then plotted on a protein standard curve. Next, 5 μL of the sample was taken, the protein concentration was determined using the BCA method (see instruction on https://www.beyotime.com, accessed on 10 December 2021), and the loading amount of each sample was calculated.

### 2.6. Western Blot 

The protein solution of the sample was taken according to the loading volume; protein loading buffer 5× was added and denatured by heating at 100 °C for 10 min. Using GenScript protein preparation gel, SDS-PAGE vertical electrophoresis and membrane transfer were performed, and after treatment with blocking solution, primary antibody, and secondary antibody, drops of ultrasensitive ECL chemiluminescent solution were added and measured after exposure. The MYOZ1 antibody was from Abcam (ab197660), and a level of GAPDH (antibody from HUABIO, ET1601-4) was used as a loading control. The working concentration for MYOZ1 and GAPDH antibodies were 0.5 and 0.2 μg/mL, respectively.

### 2.7. Design of Primers for SNP Screening 

The *MYOZ1* (accession number NC_051777) gene sequence and mRNA sequence (accession number XM_005015683) of duck (*Anas platyrhynchos*) were researched in GenBank and through Primer Premier 6.0 to design a quantitative primer (Table 2). The protein coding (CDS) region of the *MYOZ1* mRNA sequence was amplified by polymerase chain reaction (PCR) for single-nucleotide polymorphism analysis and synthesized by Nanjing Qingke Biotechnology Co., Ltd. (Nanjing, China).

### 2.8. Genotype Frequency and Gene Frequency

A total of 6 HF ducks and 6 LF ducks were selected for the polymorphism analysis of the *MYOZ1* gene. Mixed-pool sequencing was performed first, and the primers presented in Table 2 were used. The protein coding region of *MYOZ1* was expanded to a sufficient number by PCR (Applied Biosystems VeritiPro PCR, Waltham, MA, USA). In this study, a 25 μL system was used for PCR amplification. The reaction system was: 12.5 μL of 2× Taq mix, 9.5 μL of ddH_2_O, 1 μL (10 μmol/L) of upstream primer, 1μL (10 μmol/L) of downstream primer, and 1 μL of DNA template. The PCR procedure was as follows: 95 °C, 5 min, 1 cycle; 35 cycles of 95 °C, 30 s, 57 °C, 30 s, 72 °C, 32 s; 72 °C, 5 min, 1 cycle; 4 °C preservation. Samples were then sent to sequencing (by Nanjing Qingke Biotechnology Co., Ltd., Nanjing, China). The sequence alignment analysis was performed using ChromasProto to screen out the qualified SNP loci. Then, the blood DNA of 50 HF ducks and 50 LF ducks was used as the template for amplification and sequencing (performed by Nanjing Qingke Biotechnology Co., Ltd., Nanjing, China) and the genotype frequency and allele frequency were counted.

### 2.9. Statistical Analyses

SPSS 18.0 (SPSS China, Shanghai, China) was used to analyze the weight and feed conversion ratio of the samples, performing the *t*-test or one-way analysis of variance (Duncan’s test) for statistical significance of differences between different groups, and the results were expressed as mean ± standard deviation. The expression levels were tested for significance. *p* < 0.05 was considered significant, *p* < 0.01 was considered extremely significant, and *p* > 0.05 was considered not significant. ImageJ software was used for grayscale analysis. GraphPad Prism8 software was used for statistical analysis and image processing.

## 3. Results

### 3.1. Feed Conversion Ratio (FCR, Feed Intake/Weight Gain) in the Highest (HF) and Lowest Feed (LF) Converter Groups

A total of 50 each of the highest and lowest feed converters providing DNA samples for the investigation showed the average body weight and FCR in their groups (Table 3).

### 3.2. Relative Expression of MYOZ1 in the Different Feed Conversion Ratio Groups 

**Figure 1:**
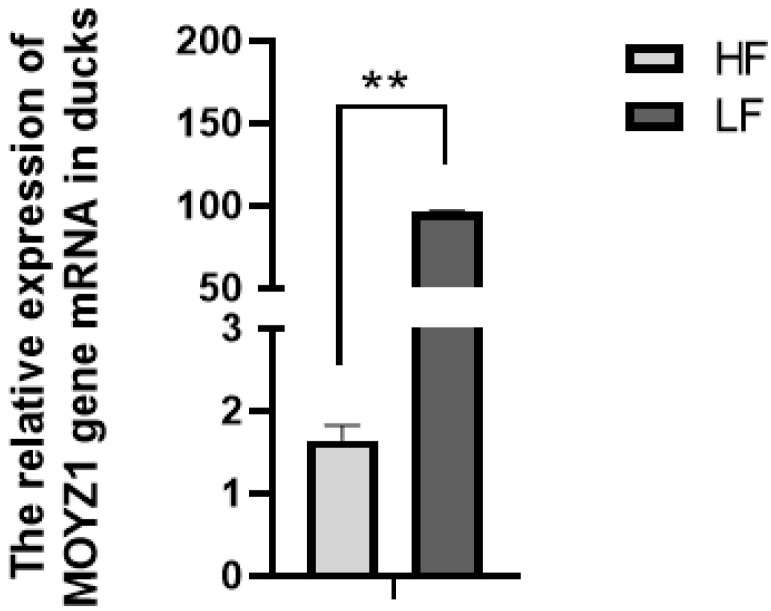
Relative mRNA expression of *MYOZ1* in HF and LF groups. ** indicates that the difference is extremely significant (*p* < 0.01).

The relative expression of *MYOZ1* in the breast muscle tissue of HF group was significantly lower than that of the LF group (*p* < 0.01) (Figure 1).

### 3.3. Relative Expression of MYOZ1 Protein in HF and LF Groups 

**Figure 2:**
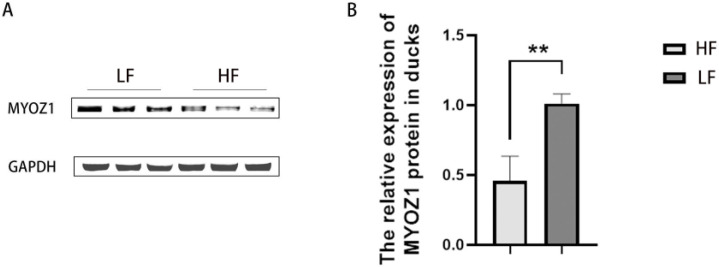
Expression of MYOZ1 and GAPDH proteins in meat ducks with high and low feed conversion ratios. (**A**) Typical map of Western blot. (**B**) Western blot gray value statistics. ** indicates that the difference is extremely significant (*p* < 0.01).

Western blot was used to detect the expression of MYOZ1 and GAPDH proteins in breast muscle tissue, and the WB results were analyzed using ImageJ software. The results are shown in Figure 2. The results showed that the expression of MYOZ1 protein in the HF group was significantly lower than that in the LF group (*p* < 0.01).

### 3.4. Analysis of Single Nucleotide Polymorphisms in MYOZ1

**Figure 3:**
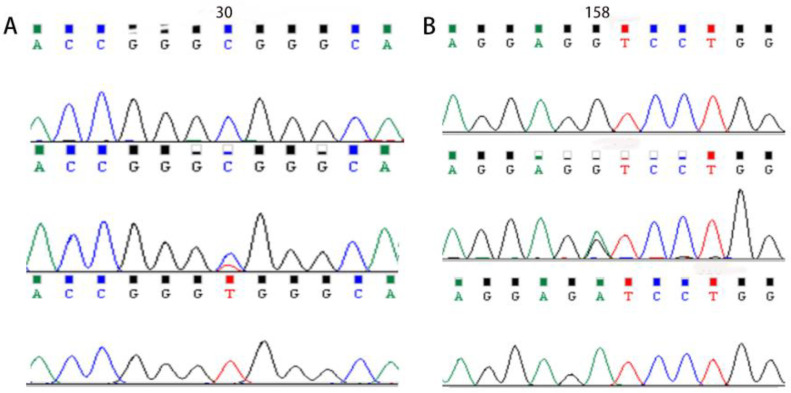
Sequencing peak map of two mutation sites in *MYOZ1*. (**A**) Sequencing peak map of the mutation site at 30 bp. (**B**) Sequencing peak map of the mutation site at 158 bp.

To verify the relationship between the SNP locus of *MYOZ1* and the feed conversion ratio, blood samples were collected from 100 ducks for DNA extraction. Three pairs of primers were designed to amplify *MYOZ1* fragments (Table 2). Two SNPs were identified using pool DNA or individual DNA sequencing, and part of the sequencing peak map is shown in Figure 3. 

As shown in Figure 3A, there were three genotypes, CC, CT, and TT, at 30 bp in the fourth exon, so this polymorphic site underwent a base C/T conversion in a point mutation; cytosine was replaced by thymine, noted as exon4 C30T. In Figure 3B, there were three genotypes, GG, GA, and AA, and a G/A transition in a base substitution mutation occurred; guanine was replaced by adenine. The location of this polymorphic site in *MYOZ1* was found at 158 bp of the fourth exon of *MYOZ1* (exon4 G158A).

### 3.5. Genetic Diversity

The genotypes of 50 HF individuals and 50 LF individuals that were successfully sequenced were counted, and the results are shown in Table 4. The genotype frequency distribution of the exon4 C30T site and exon4 G158A site in both HF and LF duck populations conformed to the Hardy–Weinberg equilibrium (*p* > 0.05). At exon4 C30T and exon4 G158A, the frequencies of CC and GG genotypes in the LF group were the highest, and the TT genotype appeared; the genotype frequencies of CC and AG were highest in the HF group. Among the groups with different feed conversion ratios, the allele frequencies at the two loci were extremely significantly different (*p* < 0.01). Therefore, we infer that the T allele and G allele may be related to a lower feed conversion ratio.

### 3.6. Feed Conversion Ratio of Different Genotypes of Meat Ducks

Among the groups with different genotypes (Table 5), at the exon4 C30T locus, the feed conversion ratio of TT genotype and CT genotype ducks was significantly lower than that of CC genotype ducks (*p* < 0.01). Therefore, it can be inferred that the T allele can be related to a lower feed conversion ratio of duck meat. At the exon4 G158A site, the feed conversion ratio of the GG genotype was significantly lower than that of the AG genotype and AA genotype (*p* < 0.01). Thus, it was determined that the G allele was significantly negatively correlated with feed conversion ratio of meat ducks. 

## 4. Discussion

Previous studies have pointed out that *MYOZ1* is involved in the structure and assembly of muscle cells, and its encoded MYOZ1 protein can bind to related proteins on the Z line, which not only affects the assembly and stability of skeletal muscle Z disks but also affects the ratio of fast muscle fiber to slow muscle fiber during the construction process [18]. In 2009, Zhu et al. obtained the calmodulin protein interacting with calsarcin-2 protein in porcine skeletal muscle using yeast two-hybrid technology, and speculated that *MYOZ1* is involved in the process of calcium ion signal transduction and muscle contraction energy transmission as well as maintaining the stability of the Z-disk structure [27]. In 2008, Su et al. took pigs as the experimental object, and the exploration showed that the *MYOZ1* gene was specifically expressed in skeletal muscle fibers [28]. In addition, this gene played a prominent role in the generation and metabolism of muscle cells, and its expression level gradually increased with the age of pigs [14]. In 2016, Ren et al. showed that the interactions between *MYOZ1* and certain matrices could lead to altered skeletal muscle composition, inducing changes in functional performance that affected muscle appearance and flavor [22]. In 2019, Luo analyzed the correlation between *MYOZ1* polymorphism and chicken slaughtering traits and found that the expression of *MYOZ1* in breast muscle first increased with time, then decreased, and finally increased again [29]. In the leg muscle, its expression level increased, so *MYOZ1* was specifically expressed in different muscle tissues of chickens. In 2020, Yoshimoto et al. engaged in an accurate assessment of muscle fiber maturity during skeletal muscle regeneration, and found that the expression of *MYOZ1* was gradually upregulated as muscle fibers matured during muscle regeneration, and the re-expression of the MYOZ1 protein was closely related to the degree of muscle fiber regeneration [30].

Two SNP loci (exon4 C30T and exon4 G158A) were found in the CDS region of *MYOZ1* in our study, and their allele frequencies were significantly different between the HF group and LF group. The mutation at C30T of exon4 results in a codon mutation from GGC to GGT at this location, and the amino acids determined by these two codons are unaltered and both encode proline. Therefore, the mutation is a synonymous mutation. The mutation at exon4 G158A results in the mutation of the codon from AGG to AGA, both of which encode arginine, and does not cause protein mutation, so the mutation is also a synonymous mutation. Because the third nucleotide in the triplet codon is replaced when the synonymous mutation occurs, which may lead to changes in mRNA shear rate and accuracy, the difference in mRNA expression between the HF group and LF group was analyzed in this experiment, and it is hypothesized that the mutation can affect the transcription of *MYOZ1*, causing the mRNA expression of HF meat ducks to be extremely significantly lower than that of LF meat ducks. In our study, the expression level of the MYOZ1 protein was also analyzed and compared using Western blotting. The results of Western blotting showed that the expression levels of the MYOZ1 protein in HF meat ducks were lower than those in LF meat ducks. This result is consistent with those observed at the DNA and RNA levels.

In general, the number of samples used in this experiment was sufficient, and the experimental data were statistically significant. Combined with existing research results on *MYOZ1*, the role and mechanism of meat duck muscle growth and development at the DNA, mRNA, and protein levels have been preliminarily explored. This experimental study showed that the relative expression of *MYOZ1* in the process of muscle growth and development was negatively correlated with the feed conversion ratio of ducks. Therefore, *MYOZ1* has a positive regulatory effect on the reduction in the feed conversion ratio, live weight gain, and even muscle growth of ducks.

## 5. Conclusions

*MYOZ1* is an important candidate gene for decreasing feed conversion ratio, and the SNP locus found was a synonymous mutation. The mRNA expression of *MYOZ1* in pectoral muscle tissue and the expression of MYOZ1 protein was negatively correlated with feed conversion ratio. In general, *MYOZ1* plays a positive regulatory role in duck muscle growth. This experimental study lays a certain theoretical foundation for further research on the mechanism of muscle growth in ducks and provides a theoretical basis for decreasing feed conversion ratio. It provides a candidate gene for meat duck breeding to promote muscle growth.

## Figures and Tables

**Table 1:** qRT-PCR primers for duck *MYOZ1* and *GAPDH.*

Name	Primers (5′→3′)	CT (°C)	Fragment Size (bp)
*MYOZ1*	Forward primer CCTGAGAGCAAAGCAGAGAAC Reverse primer CTTGCAAATAGAAAGGGTTGAAAAG	60	100
*GAPDH*	Forward primer GGTTGTCTCCTGCGACTTCA Reverse primer TCCTTGGATGCCATGTGGAC	60	165

**Table 2:** PCR primer for SNP screening of duck *MYOZ1.*

Name	Primers (5′→3′)	CT (°C)	Fragment Size (bp)
*MYOZ1-1*	Forward primer GTTGCCCTTGCTGATGTTCTG Reverse primer CGGAAGAATGAGGTTGTTACTCAAA	58	534

**Table 3:** Body weight and feed conversion ratio in the test groups.

Group	Live Weight (kg)	FCR (kg/kg)
HF	2.17 ± 0.30 ^A^	3.25 ± 0.25 ^A^
LF	2.37 ± 0.36 ^B^	2.37 ± 0.17 ^B^

Note: In the same column of data, different capital letters on the shoulder mark indicate significant difference (*p* < 0.01).

**Table 4:** Allele and genotype frequencies of *MYOZ1* SNP locus of duck.

**Site 1**	**Group**	**Sample Size**	**Genotype Frequency (N/Frequency)**			**X^2^ Value** **(*p* Value)**	**Allele Frequency**	
			**CC**	**CT**	**TT**		**C**	**T**
Exon4 C30T	LF	50	35/0.70	14/0.28	1/0.02	0.09 (0.77)	0.84	0.16
	HF	50	47/0.94	3/0.06	0/0.00	0.05 (0.83)	0.97	0.03
	LF vs. HF	*p* = 0.001						
**Site 2**	**Group**	**Sample Size**	**Genotype Frequency (N/Frequency)**			**X^2^ Value** **(*p* Value)**	**Allele Frequency**	
			**AA**	**AG**	**GG**		**A**	**G**
Exon4 G158A	LF	50	5/0.10	18/0.36	27/0.54	0.57 (0.45)	0.28	0.72
	HF	50	10/0.20	30/0.60	10/0.20	2.00 (0.16)	0.50	0.50
	LF vs. HF	*p* = 0.001						

**Table 5:** Feed conversion ratio data of different genotypes at each SNP locus.

Site	Group	Number of Individuals	FCR (kg/kg)	Effect Value (*p* Value)
Exon4 C30T	TT	1	2.50 ± 0.00 ^A^	0.0.2450 (9.68 × 10^−6^)
	CT	17	2.51 ± 0.53 ^A^	
	CC	82	2.88 ± 0.50 ^B^	
Exon4 G158A	GG	37	2.53 ± 0.38 ^A^	0.6014 (0.003905)
	AG	48	2.95 ± 0.45 ^B^	
	AA	15	3.06 ± 0.53 ^B^	

Note: In the same column of data, different capital letters on the shoulder mark indicate significant difference (*p* < 0.01).

## Data Availability

Not applicable.

## References

[1] Zhao S., Jia Y., Xu L., Ruan Y. (2016). Research progress on molecular genetic regulation of skeletal muscle growth and development in beef cattle. China Cattle Sci..

[2] Chen F., Wu P., Shen M., He M., Chen L., Qiu C., Shi H., Zhang T., Wang J., Xie K. (2019). Transcriptome analysis of differentially expressed genes related to the growth and development of the jinghai yellow chicken. Genes.

[3] Sharlo K., Paramonova I., Turtikova O., Tyganov S., Shenkman B. (2019). Plantar mechanical stimulation prevents calcineurin-NFATc1 inactivation and slow-to-fast fiber type shift in rat soleus muscle under hindlimb unloading. J. Appl. Physiol..

[4] Liu H. (2018). Transcriptome and Genome-Wide Methylation Analysis of Skeletal Muscle in Different Developmental Stages of Chinese and Foreign Pig Embryos. Ph.D. Thesis.

[5] Ou X., Li X., Zhong Z., Zhang X. (2019). The relationship between muscle fiber characteristics and muscle quality in pigs and differences between breeds and genderbvns. Xinjiang Agric. Sci..

[6] Kawai T., Yu M. (1983). The relationship between the thickness of pork muscle fibers and meat quality. Foreign Anim. Husb..

[7] Chen B., Qiu L., Wan F., Wang H., Zhang Y., Wang Z., Chen G., Chang G. (2020). Comparative analysis of SNPs and INDELs of TAP1 gene in different chicken breeds. Chin. Poult..

[8] Frey N., Richardson J.A., Olson E.N. (2000). Calsarcins, A novel family of sarcomeric calcineurin-binding proteins. Proc. Natl. Acad. Sci. USA.

[9] Gu Z., Zhu D., Li N., Li H., Deng X., Wu C. (2003). Association between single nucleotide polymorphisms of chicken Myostatin gene and skeletal muscle and fat growth. China Sci. C.

[10] Xiang L., Sun L., Jiang H. (2016). An overview of studies on the regulation of animal production performance by the myogenic determinant (MyoD) gene family. Mod. Anim. Husb. Vet. Med..

[11] Cheng B., Li L., Wang L., Zhang H. (2012). Research progress of MEF2 gene family. China Anim. Husb..

[12] Wang Z., Yang C., Liang W., Song Q., Yan D., Tian H., Bai H., Jiang Y., Chang G., Chen G. (2021). Research on the developmental characteristics of pectoral muscles in the Cherry Valley duck embryo. China Poult..

[13] Yu C., Jiang X., Du H., Li Q., Zhang Z., Qiu M., Yang C. (2020). Research and Development Trends of Breeder Genetic Evaluation and Selection Technology in Broilers. China Poult..

[14] Zhao X. (2011). A Preliminary Study on the Function of MYOZ1 Gene in Muscle Fiber Development. Master’s Thesis.

[15] Ahmad F., Gonzalez O., Ramagli L., Xu J., Siciliano M.J., Bachinski L.L., Roberts R. (2000). Identification and characterization of a novel gene (C4 or f5) located on human chromosome 4q with specific expression in cardiac and skeletal muscle. Genomics.

[16] Faulkner G., Pallavicini A., Comelli A., Salamon M., Bortoletto G., Ievolella C., Trevisan S., Kojić S., Dalla Vecchia F., Laveder P. (2000). FATZ, a filamin-, actinin-, and telethonin-binding protein of the Z-disc of skeletal muscle. J. Biol. Chem..

[17] Luo B., Xu H., Ma E., Ye F., Cui C., Zhu Q., Zhao X., Li D., Yin H., Wang Y. Chicken MYOZ1 gene expression may be regulated by MuR F1. Proceedings of the 19th Academic Symposium of the 2018 Annual Academic Conference of China Animal Husbandry and Veterinary Society Episode.

[18] Luo B., Ye M., Xu H., Ma E., Ye F., Cui C., Zhu Q., Zhao X., Yin H., Diyan L. (2018). Expression analysis, single-nucleotide polymorphisms of the MYOZ1 gene and their association with carcase and meat quality traits in chickens. Ital. J. Anim. Sci..

[19] Frey N., Frank D., Lippl S., Kuhn C., Kögler H., Barrientos T., Rohr C., Will R., Müller O.J., Weiler H. (2008). Calsarcin-2 deficiency increases exercise capacity in mice through calcineurin/NFAT activation. J. Clin. Investig..

[20] Ma J., Chai J., Shang Y., Li Y., Chen R., Jia J., Jiang S., Peng J. (2015). Swine PPAR-γ2 expression upregulated in skeletal muscle of transgenic mice via the swine Myozenin-1 gene promoter. Transgenic Res..

[21] Takada F., Woude D.L.V., Tong H.Q., Thompson T.G., Watkins S.C., Kunkel L.M., Beggs A.H. (2001). Myozenin: An alpha-actinin- and gamma-filamin-binding protein of skeletal muscle Z lines. Proc. Natl. Acad. Sci. USA.

[22] Ren R.M., Liu H., Zhao S.H., Cao J.H. (2016). Targeting of miR-432 to myozenin1 to regulate myoblast proliferation and differentiation. Genet. Mol. Res. GMR.

[23] Wang M., Li A., Zhao Z., Zhang Y., Duan M., Wang Y., Li S., Zan L. (2016). Activity of bovine MYOZ1 gene promoter. J. Northwest A F Univ. (Nat. Sci. Ed.).

[24] Arola A.M., Sanchez X., Murphy R.T., Hasle E., Li H., Elliott P.M., McKenna W.J., Towbin J.A., Bowles N.E. (2007). Mutations in PDLIM3 and MYOZ1 encoding myocyte Z line proteins are infrequently found in idiopathic dilated cardiomyopathy. Mol. Genet. Metab..

[25] Yang C., Wang Z., Song Q., Dong B., Bi Y., Bai H., Jiang Y., Chang G., Chen G. (2022). Transcriptome Sequencing to Identify Important Genes and lncRNAs Regulating Abdominal Fat Deposition in Ducks. Animals.

[26] Hu Z., Cao J., Liu G., Zhang H., Liu X. (2020). Comparative Transcriptome Profiling of Skeletal Muscle from Black Muscovy Duck at Different Growth Stages Using RNA-seq. Genes.

[27] Zhu W. (2009). Screening of Porcine Calsarcin-2 Binding Protein by Yeast Two-Hybrid Technique. Master’s Thesis.

[28] Su Y., Liu D., Song H., Cao R., Ba C., Wang H. (2008). Cloning and tissue expression profiling of the coding region of porcine Calsarcin-2 gene. J. Liaoning Med. Coll..

[29] Luo B. (2019). Identification of Polymorphic Loci of Chicken MYOZ1 Gene and Its Role in Muscle Atrophy. Master’s Thesis.

[30] Yoshimoto Y., Ikemoto-Uezumi M., Hitachi K., Fukada S.I., Uezumi A. (2020). Methods for Accurate Assessment of Myofiber Maturity During Skeletal Muscle Regeneration. Front. Cell Dev. Biol..

